# Supplementary material for: Osteoporosis-Improving Effects of Extracellular Vesicles from Human Amniotic Membrane Stem Cells in Ovariectomized Rats
Source: Int J Mol Sci. 2025 Sep 28;26(19):9503. doi: 10.3390/ijms26199503 (PMC12525326; doi:10.3390/ijms26199503)
Supplement: Supplementary file 1 [file ijms-26-09503-s001.zip › ijms-3853361-supplementary.pdf]

Supplementary Table S1. Proteome analysis (total 751 proteins)

Accession	Protein Name	Gene Name	#PSM	Percentage	UCBSC (Reference)	Remark
P09486	SPARC	SPARC	349	2.24%	0.13%	
P02452	Collagen alpha-1(I) chain	COL1A1	312	2.00%	0.51%	Collagen
P02751-1	Isoform 1 of Fibronectin	FN1	297	1.90%	3.32%	Cytoskeleton
P21333	Filamin-A	FLNA	264	1.69%	0.58%	Cytoskeleton
P08123	Collagen alpha-2(I) chain	COL1A2	214	1.37%	0.23%	Collagen
P08670	Vimentin	VIM	207	1.33%	0.74%	
P12111	Collagen alpha-3(VI) chain	COL6A3	204	1.31%	2.31%	Collagen
P12109	Collagen alpha-1(VI) chain	COL6A1	187	1.20%	0.56%	Collagen
P35555	Fibrillin-1	FBN1	180	1.15%	0.72%	
P02461	Collagen alpha-1(III) chain	COL3A1	168	1.08%	0.14%	Collagen
P60709	Actin, cytoplasmic 1	ACTB	154	0.99%	0.92%	
Q99715	Collagen alpha-1(XII) chain	COL12A1	150	0.96%	1.04%	
Q15582	Transforming growth factor-beta-induced protein ig-h3	TGFBI	150	0.96%	0.66%	
A0A804HK76	Filamin B	FLNB	149	0.95%	0.14%	
A0A804HII9	Actinin alpha 1	ACTN1	134	0.86%	0.49%	
O00469-2	Isoform 2 of Procollagen-lysine, 2-oxoglutarate 5-dioxygenase 2	PLOD2	131	0.84%	0.12%	
P02787	Serotransferrin	TF	111	0.71%	N.D.	Cytoskeleton, Iron transporter
O43707	Alpha-actinin-4	ACTN4	110	0.70%	0.50%	
P04264	Keratin, type II cytoskeletal 1	KRT1	109	0.70%	0.25%	
P68032	Actin, alpha cardiac muscle 1	ACTC1	107	0.69%	0.56%	
P07585	Decorin	DCN	103	0.66%	0.32%	
P01033	Metalloproteinase inhibitor 1	TIMP1	100	0.64%	0.03%	Collagenase inhibitor
P08253	72 kDa type IV collagenase	MMP2	99	0.63%	0.41%	
A0A7P0TA35	Protein disulfide-isomerase	P4HB	97	0.62%	0.10%	
P05121	Plasminogen activator inhibitor 1	SERPINE1	96	0.62%	0.36%	
P26038	Moesin	MSN	94	0.60%	0.39%	
P09871	Complement C1s subcomponent	C1S	91	0.58%	0.29%	
Q12841	Follistatin-related protein 1	FSTL1	91	0.58%	0.06%	
P35908	Keratin, type II cytoskeletal 2 epidermal	KRT2	90	0.58%	0.21%	
A0A1B0GVI3	Keratin 10	KRT10	89	0.57%	0.34%	
A0A7I2V3T0	CD9 molecule	CD9	16	0.02%	0.06%	Positive marker
F8VWK8	Tetraspanin (Fragment)	CD63	11	0.03%	0.04%	Positive marker
H0YEE2	CD81 molecule	CD81	20	0.03%	0.07%	Positive marker
A0A6Q8PFJ0	Lamin A/C	LMNA	23	0.15%	0.04%	Negative marker
A0A7P0TAE9	Calnexin	CANX	4	0.01%	0.01%	Negative marker

Supplementary Table S2. Lipidome analysis

Lipid classes	Class	AMSC	UCBSC (reference)
Ceramide	Cer	14.31	1.52
Diacylglycerol	DG	0.90	0.53
Monohexosylceramide	HEX1 Cer	0.18	0.38
Dihexosylceramide	HEX2 Cer	N.D	0.05
Lysophosphatidylcholine	LPC	2.37	1.77
Lysophosphatidylglycerol	LPG	0.00	N.D
Lysophosphatidylinositol	LPI	0.00	1.30
Monoglyceride	MG	1.70	0.42
Phosphatidic acid	PA	0.00	N.D
Phosphatidylcholine	PC	71.89	51.70
Phosphatidylethanolamine	PE	6.70	3.83
Phosphatidylglycerol	PG	0.00	0.87
Phosphatidylinositol	PI	0.00	10.36
Phosphatidylserine	PS	0.00	3.92
Sphingomyelin	SM	0.32	21.44
Triacylglycerol	TG	1.63	0.40
	total	100.00	98.49
